# The validity of two commercially-available sleep trackers and actigraphy for assessment of sleep parameters in obstructive sleep apnea patients

**DOI:** 10.1371/journal.pone.0210569

**Published:** 2019-01-09

**Authors:** Alexia Gruwez, Anne-Violette Bruyneel, Marie Bruyneel

**Affiliations:** 1 Chest Service, Saint-Pierre University Hospital, Brussels, Belgium and Université Libre de Bruxelles, Brussels, Belgium; 2 Department of physiotherapy, University of Applied Sciences of Western Switzerland, Geneva, Switzerland; University of Rome Tor Vergata, ITALY

## Abstract

**Objective:**

The use of activity and sleep trackers that operate through dedicated smartphone applications has become popular in the general population. However, the validity of the data they provide has been disappointing and only Total Sleep Time (TST) is reliably recorded in healthy individuals for any of the devices tested. The purpose of this study was to evaluate the ability of two sleep trackers (Withings pulse 02 (W) and Jawbone Up (U)) to measure sleep parameters in patients suffering from obstructive sleep apnea (OSA).

**Methods:**

All patients evaluated for OSA in our sleep laboratory underwent overnight polysomnography (PSG). PSG was conducted simultaneously with three other devices: two consumer-level sleep monitors (U and W) and one actigraph (Bodymedia SenseWear Pro Armband (SWA)).

**Results:**

Of 36 patients evaluated, 22 (17 men) were diagnosed with OSA (mean apnea-hypopnea index of 37+ 23/h). Single comparisons of sleep trackers (U and W) and actigraph (SWA) were performed. Compared to PSG, SWA correctly assessed TST and Wake After Sleep Onset (WASO), and U and W correctly assessed Time In Bed (TIB) and light sleep. Intraclass correlations (ICC) revealed poor validity for all parameters and devices, except for WASO assessed by SWA.

**Conclusions:**

This is the first study assessing the validity of sleep trackers in OSA patients. In this series, we have confirmed the limited performance of wearable sleep monitors that has been previously observed in healthy subjects. In OSA patients, wearable app-based health technologies provide a good estimation of TIB and light sleep but with very poor ICC.

## Introduction

The use of activity and sleep trackers has become very popular in the general population. Different devices are available that work with dedicated smartphone applications [1]. These applications are focused on activity and sleep and allow individuals to monitor their general health [2]. They could also potentially be used to reinforce patient empowerment during treatment for sleep disorders.

Sleep trackers allow users to monitor sleep parameters related to sleep quality and quantity on a daily or moment-by-moment basis [3, 4]. However, the algorithms that are used by the manufacturers to assess these parameters remain industrial secrets. Clinicians and scientists have demonstrated that the validity of sleep trackers to assess sleep in healthy individuals is limited, regardless of the type of device tested. In a previous study [5], we showed that the Withings Pulse 02 and Jawbone Up devices provide accurate information for total sleep time (TST) measurement. Jawbone Up is also able to correctly assess the time in bed (TIB) period. In another study [6], Fitbit Classic was compared to polysomnography (PSG) and shown to provide acceptable results for TST, TIB, and sleep efficiency (SE). Mantua et al. [7] compared four trackers, including Basis Health Tracker, Misfit Shine, Fitbit flex, and Withings, to Actiwatch spectrum and PSG in 40 patients. Their analysis found that the only parameter that was reliably measured for all devices was TST. SE measures were not correlated with PSG, confirming the very limited accuracy of these devices.

As the limitations of these trackers have been shown in healthy individuals, it now makes sense to ask whether the validity of sleep trackers in patients suffering from a very common sleep disorder, obstructive sleep apnea (OSA) syndrome, is also limited.

The prevalence of OSA is increasing and is closely related to the global obesity epidemic [8]. However, OSA is still largely under-diagnosed [9]. OSA is characterized by reduced airflow caused by repetitive complete or partial upper airway obstruction. Progressive respiratory effort to overcome the obstruction can lead to microarousals and oxygen desaturation that cause sleep fragmentation and increased sympathetic neural activity [10]. To date, the gold standard for diagnosis of OSA is polysomnography [11]. However, it might also be of value to use sleep trackers in OSA patients to assess, in a very simple way, changes in sleep quality and quantity after OSA treatment.

The purpose of this study was to evaluate the ability of two sleep trackers (Withings pulse 02 (W) and Jawbone Up (U)) and of one actigraph (Bodymedia SenseWear Pro Armband (SWA)) to measure sleep architecture and sleep quantity in patients diagnosed with OSA assessed during one polysomnographic recording night in a general sleep lab.

## Materials and methods

### Study design

In this prospective study, overnight PSG was performed in the sleep laboratory of CHU Saint-Pierre, Brussels in patients being evaluated for OSA. PSG was hooked up by the sleep technologist simultaneously with three other devices: two consumer-level sleep monitors (U and W) and one actigraph (SWA). The next morning, the same technologist removed the devices and PSG.

### Patient selection

From September 2016 to June 2017, 36 adults were prospectively included on a voluntary basis. Subjects were selected based on their medical history and symptoms that led us to suspect OSA. All patients were classified as “at risk” for OSA according to the STOP-BANG questionnaire [12]: the mean score was 4.3 ± 1.2, and 21/36 were at “high risk”. The remaining 15 patients scored “intermediate risk”.

All patients underwent one night of attended-PSG. No special instructions regarding sleep were given in order to guarantee that the recordings would show only “routine” behavior, but in a hospital setting.

Among 36 patients evaluated, 22 (17 men) were diagnosed with OSA, defined as exhibiting an apnea-hypopnea index (AHI) greater than 15 per hour (mean: 37± 23/ h).

The mean age of OSA-confirmed patients (n = 22) was 53 ± 13 years, and their mean body mass index was 31.4 ± 8. Mean Epworth sleepiness score (ESS) [13] was 10 ± 6. These patients suffered from numerous comorbidities: hypertension (64%), dyslipidemia (32%), depression (23%), diabetes (18%), and hypothyroidism (5%).

This study was approved by the ethics committee of CHU St. Pierre (Number: AK/15-06-67/4522P6) and all included subjects signed written informed consent.

### Consumer-level activity and sleep monitors

The Jawbone Up and Withings Pulse 02 3-axial accelerometers are still commercially available devices that are worn on the wrist. Data generated by these devices are analyzed using device-specific smartphone applications. All participants were fitted with U and W devices placed on their non-dominant wrist for one night. W was positioned near the hand and U was placed just above it, on the forearm. For this study, we used only the “sleep mode” on both devices. Prior to use, the subject’s gender, age, height, and weight were programmed into each of the devices. Data were collected through the dedicated smartphone application.

U possesses an icon that informs the user whether it is in “sleep” or “activity” mode. The user has to push on the center of the watch to switch between modes. While in “sleep” mode, the device tracks when you fall asleep based on tiny movements present during sleep, and it sends information to the Jawbone Up app via a Bluetooth 4.0 about the time needed to fall asleep, the number of awakenings, the total time of awakening after sleep onset, the TST, and the time spent in deep and light sleep [14].

W is operated using a single button on top of the device and also has a touch-sensitive screen. The touch-screen responds to both press-force and swiping gestures. If the user wants to check their heart rate or oxygen, the device must be removed from the wrist and from its case. Then, the user must place their index finger on the back of the sensor and activate the measurement with the appropriate icon. This option was not used in the present study. Similar to U, sleep and day modes using W require manual activation and the device pairs with Withings app via Bluetooth. Similar health statistics are obtained with this device [15].

Algorithms for the detection of sleep, wake, light sleep and deep sleep are not provided by the developers of either the U or the W monitor, and researchers lack reliable information for understanding the way they work to track sleep parameters.

### Actigraphy

The Bodymedia SenseWear Pro Armband is a medical actigraph that is able to evaluate sleep patterns and physical activity on consecutive days, and can also be used in OSA patients [16–19].

The SWA activity monitor was attached to an adjustable Velcro armband that was worn on the patient’s upper arm on the non-dominant side overnight. This was the same arm as was used for the W and U devices but on the upper arm rather than the forearm. We assumed that the consumer-level activity and sleep monitors and the actigraph were measuring the same body motions even if they were not located in exactly the same place. The 3 monitors were placed on the same arm and there is no reason to believe the patients underwent significant/repetitive isolated motions of their forearm without upper arm motion during sleep.

### Polysomnography

Polysomnography was performed with a polysomnograph in the sleep laboratory of CHU St. Pierre (Dream, Medatec, Belgium), and was used to record the following items: airflow using nasal prongs, pulse oximetry using an oximeter (Nonin, Minneapolis, US), thoracic and abdominal movements using piezoelectric sensors, a submental electromyogram, one anterior tibialis electromyogram, an electroencephalogram using 5 channels (C3/A1, C4/A2, FP2/A2, FP1/A1 O1/A1), right and left electrooculograms, and an electrocardiogram. Tracheal sounds were recorded using a microphone and body position was monitored with a built-in position sensor (mercury gauge) with 4 different levels. Scoring was manually conducted by a trained sleep doctor in accordance with 2012 American Academy of Sleep Medicine scoring rules [20]. Sleep was scored in sleep stages: N1, N2, N3, and rapid eye movement (REM) sleep. Stage 3 accounts for 10%-20% of sleep and REM sleep accounts for 20%-25% of sleep in normal adult populations [21].

### Sleep parameters

We compared sleep parameters for each device with PSG values (reference method) to assess the accuracy of U, W, and SWA for evaluation of sleep parameters during the night of recording, measured parameters included [11]: TIB, TST, SE, sleep latency (SL), deep sleep, light sleep (defined as sleep stage N1 and N2 on PSG) and Wake After Sleep Onset (WASO). WASO refers to periods of wakefulness occurring after sleep onset. This parameter estimates wake time periods after sleep onset, excluding the wakefulness occurring before sleep onset.

For deep sleep assessment, we used 2 comparators. Deep sleep should refer to sleep stage N3 as measured by the reference method, namely PSG. However, in the absence of information about the manufacturer’s algorithm, deep sleep, as measured by the sleep trackers, could also refer to other PSG parameters, such as the sum of N3+REM. This hypothesis has been tested by Mantua et al. They described a positive correlation between the deep sleep measurement of W with the “N3+REM” determination by PSG [7]. Both definitions have been used in the present study: Deep Sleep A refers to N3 and Deep Sleep B refers to N3+ REM.

SWA does not measure deep and light sleep.

### Statistical methods

Different stages characterized the statistical analysis: 1) comparisons between several measurement methods (PSG vs. U vs. SWA vs. W), and 2) validity analysis for TST, TIB, SE, SL, light sleep, deep sleep A, deep sleep B, WASO, and awakenings. The statistical analysis included descriptive statistics with mean and standard deviation (SD) to describe all methods. The Wilcoxon signed rank test for paired samples was applied for comparisons between PSG, U, SWA, and W methods. A threshold value of *p* < 0.05 was adopted for ruling out non-significant differences.

We calculated the intra-class correlation coefficient (ICC) and used the Bland and Altman limits of agreement (LOA) method to assess agreement between the methods. A graphic plot was produced to highlight differences between each pair of measurements in relation to the mean of each pair. LOA 95% (mean difference ± 2 SD) values are superimposed on this plot. For the TST parameter, the 30 minutes clinical acceptance range between commercial tracker and PSG previously described by Meltzer was also added to the Bland and Altman plot [22]. This allows good visual representation of the validity between two methods of observation for each parameter. An ICC greater than 0.75 was considered “good validity”, an ICC between 0.50 and 0.75 indicated “moderate validity, and less than 0.50 indicated “poor validity” [23]. All data were analyzed with Statistica (v.10. Statsoft) or R software (Version 3.1.1) with the ICC package (Facilitating Estimation of the Intraclass Correlation Coefficient 2.3.0. Matthew Wollak, URL address: https://cran.r-project.org/web/packages/ICC/index.html).

## Results

### Device failures

The failure rate of the recording devices was 0% for PSG, 4.5% for SWA, 9% for W, and 0% for U. For W, the failures where related to tracker malfunction (50%) or Software dysfunction (interruption of the device during the night) (50%). For SWA, 100% of failures were due to failure of data download.

### Sleep parameters

Compared to PSG, SWA showed non-significant differences in TST and WASO measurements (p>0.05, Table 1). However, TIB was shorter (p = 0.0032), as was SL (p = 0.0004), and SE was better (80% vs 67%, p = 0.0009), while the number of detected awakenings was much lower (p = 0.0006) for SWA compared to PSG.

**Table 1:** Single comparisons of sleep parameters obtained by polysomnography (PSG) vs sleep trackers.

N = 22	PSG	SWA	*P value*	U	*P value*	W	*P value*
TST (min)	344±92	358±90	0.1368	407±60	***0*.*006***	431±68	***0*.*0007***
TIB (min)	519±34	446±86	***0*.*0032***	522±45	0.1774	524±122	0.1445
SE (%)	67±18	80±14	***0*.*0009***	78±13	***0*.*0004***	84±11	***0*.*0007***
SL (min)	82±82	18±26	***0*.*0004***	44±31	***0*.*0097***	12±5	***0*.*0003***
Light sleep (min)	190±85	-	NA	163±69	0.1057	239±54	0.1119
Deep sleep A (min)	83±51	-	NA	243±74	***0*.*00001***	192±84	***0*.*00002***
Deep sleep B (min)	70±33	-	NA	243±74	***0*.*000007***	192±84	***0*.*00001***
WASO (min)	100±90	79±76	0.0698	56±57	***0*.*0057***	49±51	***0*.*0007***
Awakenings (n)	29±15	9±6	***0*.*0006***	3±2	***0*.*0001***	4±3	***0*.*0002***

U and W accurately assessed TIB and light sleep (p>0.05). None of the test devices accurately measured SE, SL, or awakenings. For deep sleep, regardless of the chosen comparator, A or B, U and W could not estimate it properly (Table 1).

Intraclass correlations (ICC) revealed poor validity for all parameters and devices. The best value was obtained for WASO assessed by SWA, where moderate validity was obtained: ICC value of 0.5 (Table 2). No valid correlations could be obtained for U and W (ICC values < 0.45, Table 2). As ICC values were all <0.75, no standard error measurements or minimal detectable changes were calculated.

**Table 2:** Intraclass correlations between PSG and SWA, PSG and U, and PSG and W.

	ICC		
N = 22	PSG vs. SWA	PSG vs. U	PSG vs. W
TST (min)	0.41	0.27	0.13
TIB (min)	0.15	0.27	0.09
SE (%)	0.42	0.38	0.15
SL (min)	0.14	0.08	-0.003
Light sleep (min)	NA	0.03	-0.31
Deep sleep A (min)	NA	-0.01	0.1
Deep sleep B (min)	NA	0.05	0.06
WASO (min)	***0*.*5***	0.37	0.44
Awakenings (n)	0.01	0.01	-0.01

**Fig 1:**
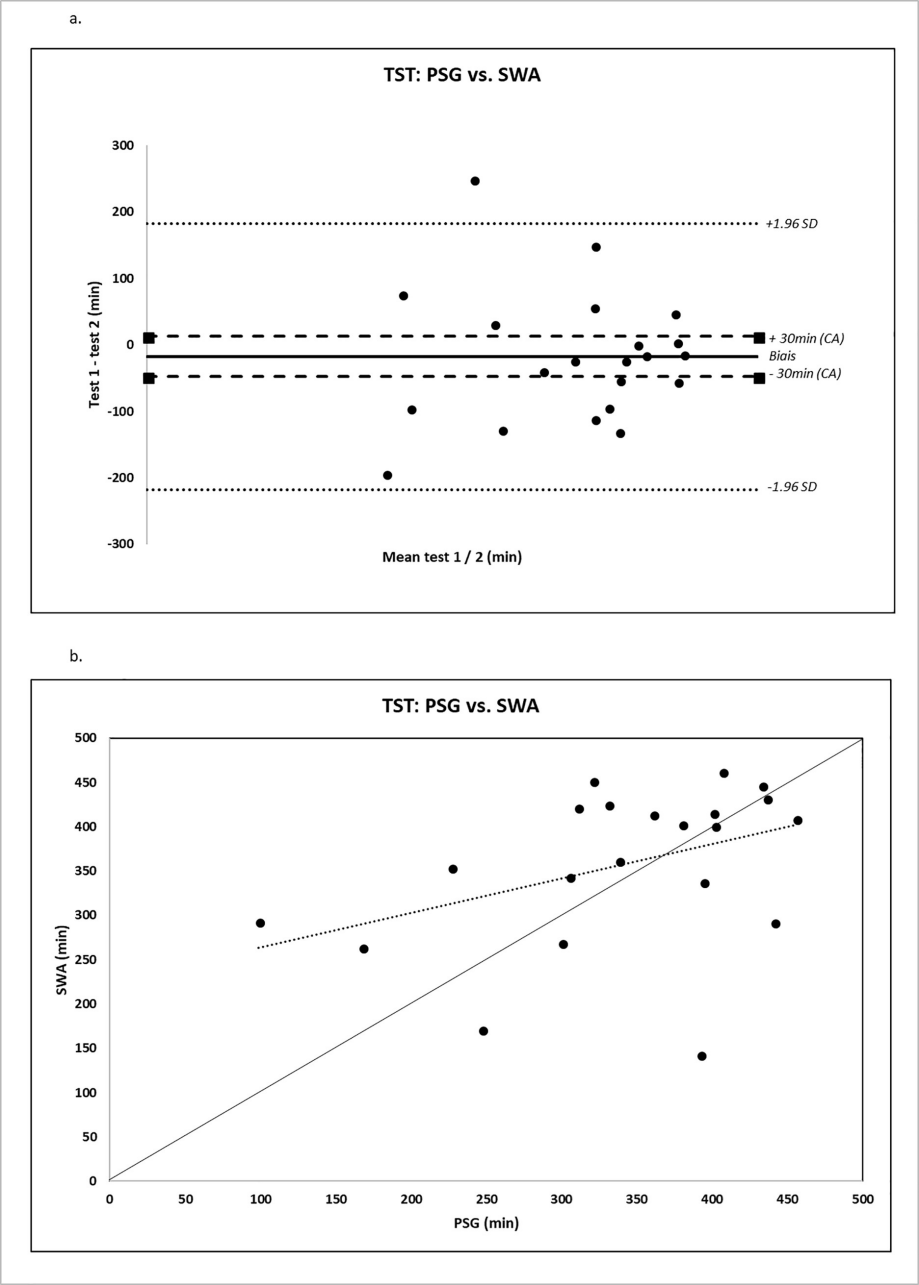
TST: PSG vs. SWA. Fig 1A. represents Bland-Altman plots of differences between measurements of total sleep time (TST), expressed in minutes, as measured by PSG (Polysomnography) and by the Bodymedia SenseWear Pro Armband (SWA). Fig 1B. shows the agreement between PSG and SWA for TST parameter. A systematic effect is observed because values are not uniformly distributed around the identity line (line with a 45° slope).

**Fig 2:**
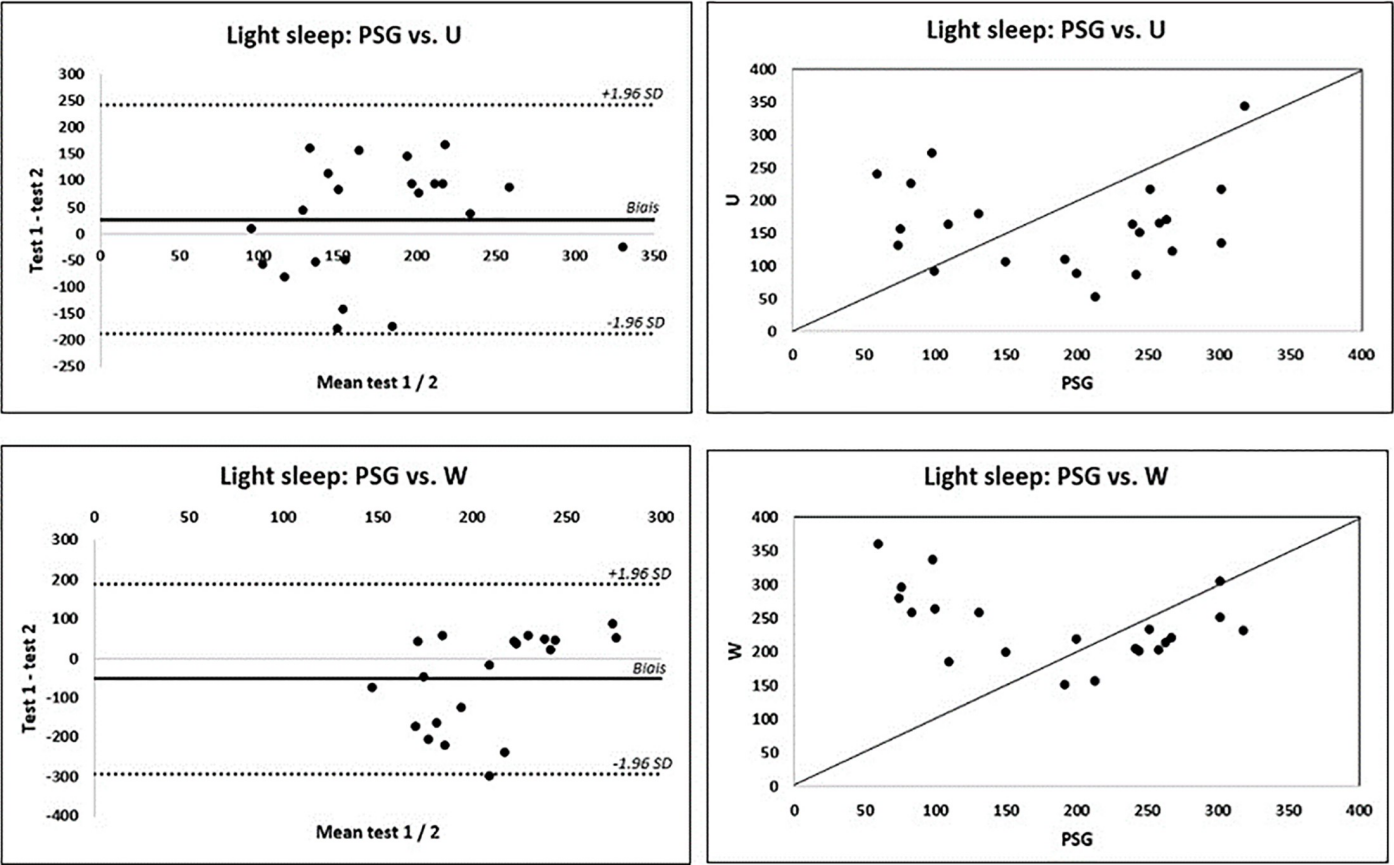
Reliability for light sleep parameter. Fig 2A. (left) represents Bland-Altman plots of differences between measurements of light sleep as measured by PSG (polysomnography), expressed in minutes, and by the activity monitors (U shows an underestimation of 27 min, in contrast to W, which shows an overestimation of 52 min). W: Withings pulse 02, U: Up Move Jawbone. Fig 2B. (right) shows the agreement between PSG and activity monitors (U and W) for the light sleep parameter. A systematic effect is observed because values are not uniformly distributed around the identity line (line with a 45° slope).

Using Bland-Altman plots, important over/underestimations were obtained for all devices and all parameters, with SWA performing globally better. For TST and WASO assessment, biases were lower for SWA than for U and W (-17 vs -63 vs -87 min (TST) and 21 vs 44 vs 55 min for WASO, respectively) (Fig 1). In contrast, U and W performed better for TIB (bias of -3, -5, + 73 min, respectively). Sleep efficiency was overestimated by the 3 devices in the same proportions (-14% for SWA, -12% for U, and -17% for W). Light sleep was underestimated by U (27 min) and overestimated by W (-52 min, Fig 2). Results are summarized in Table 3.

**Table 3:** Bland-Altman plot bias and limits of agreement results for sleep parameters for all devices.

	PSG vs. SWA		PSG vs. U		PSG vs. W	
N = 22	Mean difference (bias)	LOA95% (2SD)	Mean difference (bias)	LOA95% (2SD)	Mean difference (bias)	LOA95% (2SD)
TST (min)	-17	±200	-63	±176	-87	±208
TIB (min)	73	±208	-3	±97	-5	±243
SE (%)	-14	±30	-12	±33	-17	±38
SL (min)	67	±153	38	±167	66	±165
Light sleep (min)		NA	27	±215	-52	±240
Deep sleep A (min)		NA	-160	±182	88	±176
Deep sleep B (min)		NA	-173	±135	-121	±168
WASO (min)	21	±168	44	±163	55	±138
Awakenings (n)	21	±34	26	±31	26	±32

## Discussion

This is the first study to assess the validity of sleep trackers in OSA patients. In this series of OSA patients recorded for a single night in a hospital setting concomitantly by two commercially-available sleep monitors and two medically-validated tools (PSG and SWA), we observed that wearable app-based health technologies give a fair estimation of TIB and light sleep but with very poor intraclass correlation.

In a recent study, Toon et al. [24] showed, in 78 children and adolescents suffering from snoring and OSA, that U gave accurate data for SL, TST, WASO, and SE measures. Overestimation of TST was 9 minutes, contrary to the 63-minute difference observed in our series. SE was also quite well measured, with a mean difference of 1.8%. U seems to generally work better in adolescents, as shown by de Zambotti et al. [25], who described similar results in 65 healthy adolescents. Their results showed that U overestimated sleep by 10 minutes and SE by 1.9%. The discrepancies observed in adult and children/adolescent populations could originate from the differences in sleep architecture between these populations. Indeed, even in the presence of OSA, sleep duration, sleep efficiency, and sleep continuity are better preserved in children/adolescents than in the adult population [26–28].

A recent interesting study [29], performed in insomniacs and good sleepers, established the validity of the Fitbit Flex device for measuring sleep parameters. Compared to PSG, a slight overestimation of TST (33 min) and of SE (8%) was observed in insomniacs. The best ICC was obtained for TST (0.88), but the concordance for SL, SE, and WASO was poor, such that the authors concluded that the sleep tracker could be useful for completion of sleep diaries in insomniacs but could not replace PSG or actigraphic measures.

Regarding actigraphy, our results confirmed good agreement for TST and WASO, but with moderate validity, according to ICC, and only for WASO. The good agreement reported by Sharif et al. [30] for SE was not observed in our series, maybe because of the small size of the study population.

There are some limitations related to the present work. The small size of the population should lead to cautious interpretation of our results, which should be confirmed in a larger OSA population. Generalization of results is thus questionable and further studies must be performed to confirm the present findings. Also, the severity of OSA in this study population was quite high (AHI of 37 +/-23) which could also influence the results.

Wearable devices are mainly used in an unattended setting. One single night of recording in a sleep lab is not a good reflection of usual sleep in all patients. Therefore, home studies should be carried out in order to effectively validate their usefulness in routine settings.

We can conclude that the present study confirms, in patients suffering from OSA, the limited performance of wearable sleep monitors. Based on the high prevalence of undiagnosed OSA in the general population, it is clear that OSA patients, unaware of their condition, are going to use such devices, with a risk of misleading individuals who rely on recorded data. To improve the performance of such devices, technology manufacturers should develop algorithms in collaboration with clinical or research enterprises. For example, sleep monitors could bring additional information to sleep diaries in insomniacs, or may play a role to better understand sleep schedules in shift workers. One interesting avenue for future development of consumer sleep trackers could be based on electroencephalographic readings [31], leading to the collection of numerous physiologic data to assess sleep structure, and, thus, potentially to more accurate sleep data. These would work via a face mask or headphones. No medical research has been published on these devices. Further studies are needed to better determine the role of sleep trackers in patients suffering from common sleep disorders.

## Supporting information

S1 File Raw data for each measuring device for TST, TIB, WASO, REM, Deep Sleep, Light Sleep, number of awakenings, sleep latency and sleep efficiency parameters. (XLSX)

## Abbreviations

PSG: polysomnography
W: Withings pulse
U: Up Move Jawbone
SWA: Bodymedia SenseWear Pro Armband
TST: Total Sleep Time
TIB: Time in Bed
SE: Sleep Efficiency
SL: Sleep Latency
N3: sleep stage 3
REM: rapid eye movement sleep
WASO: wake after sleep onset
SD: standard deviation
